# REM sleep behavior disorder was associated with Parkinson’s disease: a community-based study

**DOI:** 10.1186/s12883-016-0640-1

**Published:** 2016-08-02

**Authors:** Jian-Fang Ma, Miao-Miao Hou, Hui-Dong Tang, Xiang Gao, Liang Liang, Li-Fang Zhu, Yi Zhou, Sheng-Yu Zha, Shi-Shuang Cui, Juan-Juan Du, Gen Li, Jun Liu, Sheng-Di Chen

**Affiliations:** 1 Department of Neurology and Institute of Neurology, Ruijin Hospital affiliated to Shanghai Jiao Tong University School of Medicine, Shanghai, 200025 People’s Republic of China; 2 Department of Nutritional Sciences, The Pennsylvania State University, University Park, PA 16802 USA; 3 Malu Medical Center, Jia Ding district, Shanghai, 201800 People’s Republic of China

**Keywords:** Community-based study, Parkinson’s disease, Prevalence, REM sleep behavior disorder

## Abstract

**Background:**

Our study was aimed to validate a modified RBD (REM sleep behavior disorder) single question (RBD1Q-C), study the prevalence of probable RBD (*p*RBD) in a rural community based on RBD1Q-C and investigate the association between *p*RBD and Parkinson’s disease (PD).

**Methods:**

The validation study of RBD1Q-C included 32 Chinese participants (14 idiopathic RBD patients and 18 controls). All participants underwent a polysomnogram (PSG). We then conducted a door-to-door survey to estimate the prevalence of *p*RBD assessed by RBD1Q-C, and its association with PD among 19614 residents who lived in Malu community of Shanghai, China.

**Results:**

RBD1Q-C demonstrated a high sensitivity of 100 %, a moderate specificity of 55.6 %. The agreement between RBD1Q-C and PSG-based RBD diagnosis was good (k = 0.552). PPV of the RBD1Q-C was 63.6 % and NPV was 100 %. The prevalence of *p*RBD in Malu community was 4.9 %. In people over 50 years old, presence of *p*RBD was significantly associated with increased risk of having PD (odds ratio = 2.61, 95 % CI: 1.56–4.39).

**Conclusion:**

RBD1Q-C was shown to be a useful screening tool. Based on the RBD1Q-C, we found that *p*RBD was not rare in Chinese rural population and associated with odds of PD, calling for more attention from patients, caregivers and physicians.

**Electronic supplementary material:**

The online version of this article (doi:10.1186/s12883-016-0640-1) contains supplementary material, which is available to authorized users.

## Background

Rapid eye movement (REM) sleep behavior disorder (RBD) is a parasomnia characterized by dream enactment behaviors in the setting of increased electromyographic activity during REM sleep [1]. These dream enactment behaviors occur almost always in the setting of an upsetting dream and present as sleep talking, shouting, and vigorous or elaborate body movements including punching, kicking, sitting up, and even falling out of bed [2]. Recent studies found that RBD may represent an early clinical manifestation of a developing neurodegenerative disorders, mostly synucleinopathies such as PD (Parkinson’s disease), MSA (multiple system atrophy) and DLB (Lewy Body Dementia) [3–6]. Reliable, accurate methods for identifying RBD may offer a window for early intervention.

Polysomnographic (PSG) is required for the diagnosis of RBD according to the third edition of the International Classification of Sleep Disorders (ICSD-III). However, PSGs are time and resource consuming. The REM Sleep Behavior Disorder Single-Question Screen (RBD1Q) was developed and validated to be an easily applicable and short diagnostic screening tool [7]. The RBD1Q can detect RBD with 94 % sensitivity and 87 % specificity in a well-characterized, clinic-based cohort of RBD patients and controls.

Because of concerns about the specificity, RBD1Q does not screen for sleep talking or sleep yelling. While the single item analysis of Innsbruck RBD inventory (RBD-I) revealed that sleep-related vocalization (sleep talking not included) had a good specificity of 94.2 % and AUC (0.804) [8]. In consideration of a subset of patients for whom this is the only manifestation, we modified RBD1Q into a Chinese version of RBD1Q (RBD1Q-C) with the inclusion of sleep screaming and sleep yelling in order to obtain a higher sensitivity for the screening purpose. In addition, as the prevalence of RBD and the association of RBD with neurodegenerative diseases has not been well studied in Chinese general population [9–11], we aimed in this study to (1) validate the modified RBD1Q (RBD1Q-C); (2) estimate prevalence of probable RBD (*p*RBD) in a population-based survey by using RBD1Q-C as a screening tool; and (3) examine whether *p*RBD based on RBD1Q-C was associated with Parkinson’s disease.

## Methods

### Screening question

RBD1Q was translated and back-translated by a senior neurologist and a professional translator. Then we included sleep shouting or yelling into the question, named as Chinese version of RBD1Q (RBD1Q-C). RBD1Q-C consists of a single question, answered “yes” or “no,” as follows “Did you have the following conditions (or have you ever been told by your husband or wife) that you shout, yell, move your arms or legs in response to your dream contents, even fallen off your bed?” (Additional file 1). This question was designed to be self-administered, with participation by caregivers encouraged.

### Participants

#### For validation study of RBD1Q-C

Patients referred to the Departments of Respiratory and Neurology for sleep complaints were recruited in this study based on consequential sampling. People who were unwilling or unable to participate (such as hearing problems) were excluded. People were asked to complete the RBD1Q-C before PSG study after consent. Therefore they did not know their final diagnosis when they completed the questionnaire. All RBD patients had RBD diagnosis confirmed by history and PSG according to the ICSD-III. And patients were classified as the control group who did not meet the RBD diagnosis based on PSG and history. Patients who failed to attain REM sleep on their PSG were excluded from the study.

#### For pRBD prevalence study

This door-to-door study was conducted in Malu community of Jiading district which is located in north-west rural area of Shanghai from April to October 2013. According to the censorship, there are 45353 residents aged ≥20 years. About 31202 (68.8 %) residents completed RBD1Q-C. *p*RBD diagnosis was based on RBD1Q without PSG study. Therefore, we used *p*RBD instead of RBD in our study.

#### For PD ascertainment

We did a two-step door-to-door study to screen PD patients in people aged 50 years or older in Malu community to examine whether RBD was associated with PD. In the first step, local doctors from medical center of Malu community had one-day clinical teaching course of evaluating parkinsonism and essential tremor by movement disorders specialists from the Department of Neurology, Rui Jin Hospital affiliated to Shanghai Jiao Tong University School of Medicine. This clinical teaching course included video presentation of symptoms of parkinsonism and essential tremor and how to retrieve the history. Under the supervision of movement disorder specialist, local doctor was considered qualified when they demonstrated competency to do the clinical examination of tremor, bradykinesia, rigidity and gait. After being trained, these local doctors visited residents ≥50 years from door to door and reported possible parkinsonism cases to the movement disorder specialists. In the second step, movement disorder specialists revisited these possible parkinsonism cases to made the final diagnosis which included imaging studies, routine exclusion of other parkinsonian disorders such as multiple system atrophy, progressive supranuclear palsy, or cortico-basal degeneration, etc. The diagnosis of PD patients was based on the United Kingdom Parkinson’s Disease Society Brain Bank Clinical Diagnostic Criteria.

### Statistical analysis

Analysis was carried out by using IBM SPSS Statistics Version 17.0 (SPSS, Chicago, IL). For validation study of RBD1Q-C, demographic feature of participants were compared by student *t* test and *χ*^2^ test. The psychometric properties of the RBD1Q-C were assessed by calculating the sensitivity, specificity, positive predictive value (PPV) and negative predictive value (NPV) for each threshold with a 95 % confidence interval (CI). The diagnostic value of RBD1Q-C was calculated by the area under the curve (AUC). The agreement (k coefficent) of RBD1Q-C with PSG was calculated. k values were used to categorize results as very good (0.81–1.0), good (0.61–0.8), moderate (0.41–0.6), fair (0.21–0.4) or poor (0–0.2). The Fisher exact test was used to compare the detection rate between the RBD1Q-C and PSG-based RBD diagnosis and a *p* value <0.05 was considered significantly different.

To estimate the association between *p*RBD and PD, we used logistic regression model to calculate odds ratio (OR) with 95 % confidence interval (95 % CI), according to *p*RBD status, adjusting for age and sex.

## Results

### Validation of RBD1Q-C

A total of 139 participants with sleep complaints were enrolled (26–87 year, 47 female and 92 male). These patients were not on medication associated with RBD such as SSRI antidepressant. People on benzodiazepine were asked to stop medication for at least one week to perform the PSG test. About 46 subjects were excluded because of lack REM sleep during v-PSG or technical problems (10 PD with RBD, 1 iRBD, 12 OSAS, 8 insomnia, 5 RLS, 3 epilepsy, 7 others). Among 93 remaining subjects, 36 were diagnosed as RBD (20 PD with RBD, 2 MSA with RBD, and 14 idiopathic RBD) and 57 non-RBD (2 PD without RBD, 4 Restless legs syndrome, 26 OSAS (10 severe OSAS), 8 Epilepsy, 1 subdural hemorrhage, 1 dementia with insomnia, 8 insomnia, and 8 others). We included totally 32 subjects into our validation study: 14 idiopathic RBD patients and 18 non-RBD controls (1 subdural hemorrhage, 1 dementia with insomnia, 8 insomnia and 8 others). Mean RBD patient age was 64.07 ± 4.62 years old, in comparable to non-RBD group (60.39 ± 13.00) (*p* = 0.32). About 50 % of RBD patients were men, compared to 66.7 % of controls (*p* = 0.47).

In 14 RBD patients, 14 answered “yes” to RBD1Q-C, translating to a sensitivity of 100 % (95 %CI: 73.2–100 %) and 10 out of 18 non-RBD answered “no” to RBD1Q-C, translating to a specificity of 55.6 % (95 % CI: 31.3–77.6 %). Area under the receiver operating characteristic (ROC) curve was 0.778 (95 % CI: 0.615–0.940). The agreement between RBD1Q-C and PSG-based RBD was good (k = 0.552). The positive predictive value (PPV) of the RBD1Q-C was 63.6 % (95 % CI: 40.8–82.0 %) and negative predictive value (NPV) was 100 % (95 % CI: 65.5–100 %).

### Prevalence of *p*RBD

There were 1521 of 31202 participants who answered “yes” to RBD1Q-C. Among the 1521 residents, 612 were men (40.2 %), 909 were women (59.8 %), the mean age was 66.53 ± 11.48 years (range 20–99). The crude prevalence of *p*RBD (diagnosis of *p*RBD was based on RBD1Q without PSG confirming) in Malu community was estimated to be 4.9 % (95 % CI: 4.6–5.1 %), significantly higher for women than men (5.6 vs. 4.1 %, *p* < 0.001). For both men and women, the prevalence of *p*RBD increased with age and reached the peak in the 70–79 age population (Table 1).

**Table 1:** Population and prevalence of RBD by age and gender in Malu community

Age(yr)	Men		Women		Total	
	*n*	%	*n*	%	*n*	%
Population						
20–49	5779	18.5	5809	18.6	11588	37.1
50–59	3108	10.0	3073	9.8	6181	19.8
60–69	3372	10.8	3698	11.9	7070	22.7
70–79	1625	5.2	1779	5.7	3404	10.9
80–	1173	3.8	1786	5.7	2959	9.5
Total	15057	48.3	16145	51.7	31202	100.0
Prevalence of RBD						
20–49	40	0.7 95 % CI: 0.5–0.9	53	0.9 95 % CI : 0.7–1.2	93	0.8 95 % CI:0.6–1.0
50–59	129	4.2 95 % CI: 3.5–4.9	183	6.0 95 % CI: 5.1–6.8	312	5.0 95 % CI:4.5–5.6
60–69	201	6.0 95 % CI:5.2–6.8	304	8.2 95 % CI: 7.4–9.1	505	7.1 95 % CI: 6.6–7.8
70–79	168	10.3 95 % CI :8.9–11.9	222	12.5 95 % CI:11.0–14.1	390	11.5 95 % CI:10.4–12.5
80–	74	6.3 95 % CI:5.0–7.8	147	8.2 95 % CI:7.0–9.5	221	7.5 95 % CI:6.5–8.4
Total	612	4.1 95 % CI:3.8–4.4	909	5.6 95 % CI:5.3–6.0	1521	4.9 95 % CI:4.6–5.1

### RBD and PD

Totally 19614 people over 50 years old answered RBD1Q-C questionnaire (1428 answered “yes” and 18186 answered “no”) and examined by local doctors and further movement disorders specialists for PD diagnosis. After first screen, 124 subjects were diagnosed as parkinsonism by local doctors. Movement disorders specialist revisited 124 subjects and made the final diagnosis of PD in 97 subjects. Prevalence of PD was 1.2 % among those with *p*RBD and 0.4 % among those without *p*RBD (age-and sex-adjusted OR = 2.61, 95 % CI: 1.56–4.39, *P <* 0.001).

## Discussion

Our study demonstrated that RBD1Q-C was reliable to screen potential RBD in large epidemiological study. The prevalence of *p*RBD diagnosed by RBD1Q-C was 4.9 %, higher in women than men. *p*RBD was associated with PD in people over 50 years.

Regarding to the importance of RBD in synucleinopathies and the difficulty to perform PSG, a few RBD screening questionnaires have been designed to facilitate population-based studies which included RBD1Q-C, REM sleep behavior disorder screening questionnaire (RBDSQ), RBDQ-HK (Hong Kong), Mayo Sleep questionnaire (MSQ) and Innsbruck RBD inventory (RBD-I) [8, 12–14]. So far, RBDSQ and RBDQ-HK have been validated in China [15, 16]. RBD1Q-C in our study which included of sleep vocalization in RBD1Q showed a good consistency with “golden” criteria in diagnosing RBD patients, high sensitivity (100 %) and moderate specificity (55.6 %) which was comparable to RBD1Q and other screening questionnaires (Table 2). In addition, RBD1Q-C was short and easy-to-use in large/broad epidemiologic surveys.

**Table 2:** Characteristics of six validated screening tools of RBD

Scale	REM Sleep Behavior disorder Screening questionnaire (RBDSQ) [12]	Validation of RBDSQ in China [16]	Mayo Sleep Questionnaire (MSQ) [17]	REM Sleep Behavior Disorder Questionnaire–Hong Kong (RBDQ-HK) [13]	Validation of RBDQ-HK in east China [15]	RBD Single-Question Screen (RBD1Q) [7]	Innsbruck REM Sleep Behavior Disroder Inventory (RBD-I) [14]	Chinese version of RBD1Q (RBD1Q-C)
No. of items	13 items	-	1 screening question on RBD (plus 5 additional questions if screened positive)	13 questions (Or use the Factor 2 behavioral subscale, which contains 7 questions)	-	1 question	5 questions	1 question
Informants	Patient, bed partner encouraged	-	Bed partner only	Patient and/or bed partner	-	Patient, spouse/Caregiver encouraged	Patient, bed partner encouraged	Patient, bed partner encouraged
Validation study								
Study cases	*n* = 54 Idiopathic RBD RBD co-morbid with narcolepsy	*n* = 63 Idiopathic RBD RBD associated with PD	*n* = 81 RBD co-morbid with cognitive impairment/neurodegenerative diseases	*n* = 107 Idiopathic RBD RBD co-morbid with narcolepsy/neurodegenerative diseases	*n* = 115 Idiopathic RBD, RBDassociated with PD, RBD like disorder	*n* = 242 Idiopathic RBD	*n* = 70 Idiopathic RBD RBD co-morbid with narcolepsy/neurodegenerative diseases	*n* = 14 Idiopathic RBD RBD co-morbid with neurodegenerative diseases
Study controls	Sleep patients (*n* = 160) Healthy subjects (*n* = 133)	Sleep patients (*n* = 78) PD patients without RBD (*n* = 35) Healthy subjects (*n* = 50)	Patients mostly with cognitive impairment/Parkinson’s disease (*n* = 66)	Sleep patients (*n* = 86) Psychiatric patients (*n* = 6) Healthy subjects (*n* = 15)	Sleep patients(*n* = 124) Patients with neurogenerative disorder(*n* = 74) Healthy subjects(*n* = 12)	Sleep patients and healthy control (total *n* = 242)	Sleep patients(total = 140)	Sleep patients and healthy control (total *n* = 32)
Diagnostic methods	RBD patients & sleep patients: PSG. Healthy subjects: Medical history only, no PSG	PSG	PSG	PSG	PSG	PSG	PSG	PSG
Psychometric properties								
Cut-off	5/13	5/13	-	19/100 for total scale and 8/70 for Factor 2 subscale	17/100 for total scale and 7 or 8/70 for Factor 2 subscale	-	0.25 (number of positive symptoms divided by number of answered questions)	-
Sensitivity/Specificity	96 %/56 %	92.1 %/81.2 %	98 %/74 %	82.2 %/86.9 % 87.9 %/81.3 %(subscale)	85 %/81 % 90 %/82 %(subscale)	93.8 %/87.2 %	91.4 %/85.7 %	100 %/55.6 %

We reported a relatively high *p*RBD crude prevalence of 4.9 % by RBD1Q-C in comparison to other population-based RBD prevalence studies (Table 3). This might be due to the fact that *p*RBD in our prevalence study was diagnosed by RBD1Q-C instead of PSG. Several previous studies reported a comparative low prevalence of PSG as they enrolled relatively severe RBD cases, for example, the United Kingdom study investigated violent behavior during sleep and the Hong Kong study examined sleep-related injury. Interestingly, a recent study reported a prevalence of probable RBD based RBDSQ or RBDHK as high as 4.6 % which was similar to our results [17]. In addition, another finding in our study was that the prevalence of pRBD was higher in women than men, which was in contrast to the clinical finding that RBD were more common in men than women [18, 19]. It was possible that women might have a less severe form of RBD [20], and therefore less likely to be referred to a sleep clinics. Another possibility is that some *p*RBD cases screened by RBD1Q-C might be RBD mimics. Women were more likely suffer from some RBD mimics such as insomnia [21].

**Table 3:** Community-based prevalence studies of RBD

References	Year	Country	Subjects	Diagnostic methods	Prevalence of RBD	95 % CI
Chiu et al [10]	2000	Hong Kong	A representative sample of 1034 elderly, aged 70 years or above	Screening question, and those who had positive response underwent sleep studies	0.38 %	0.01 % to 0.76 %
Ohayon et al [9]	1994–2000	Finland, Germany, Italy, Portugal, Spain and the United Kingdom	A random stratified sample of 19,961 participants from the general population, 15 years and older	telephone interview	1.7 %	1.5 % to 1.8 %
Kang et al [11]	2010	Korea	Among 696 random sampling subjects aged 60 years or older, 348 subjects underwent vPSG	vPSG	2.01 %	0.54 % to 3.49 %
Mahlknecht et al [17]	2015	Caucasian population (Bruneck Study Cohort)	456 participants without Parkinson’s disease 456 participants without Parkinson’s disease 456 participants without Parkinson’s disease, 456 participants without PD aged 60 years or older	RBDSQ RBD-I	4.6 % 7.7 %	3.0 % to 7.0 % 5.6 %–10.5 %
Our study	2013	China	A door-to-door survey including 31202 residents	RBD1Q-C	4.9 %	4.6 % to 5.1 %

In our study, we found a significant association between PD and possible RBD based on RBD1Q-C screening. This finding was consistent with several other studies. Schenk et al. reported that 11 of 29 idiopathic RBD (iRBD) subjects (38 %) developed parkinsonism nearly 4 years after the diagnosis of iRBD and 13 years after the onset of RBD symptoms [4]. In a second series, 26 of 93 iRBD patients (28 %) developed PD, DLB or MSA after a mean follow-up of 5 years [22]. Iranzo et al. reported that 31 of 44 iRBD patients (70.4 %) developed a defined synucleinopathy disorder after a mean follow-up of 10.5 years [23]. These results supported the hypothesis of Braak and colleagues, which suggested that the preclinical stages 1 and 2 of PD start at the olfactory amd medullary area of the brainstem [24].

Some limitations should be noted. Firstly, for the validation study of RBD1Q-C, the number of subjects recruited was small and thus may overestimate the sensitivity, specificity, PPV and NPV. In addition, subjects who know RBD might affect their response to the RBD1Q-C [25]. For prevalence study, our population was from East China, thus not representing the whole country. Also, RBD1Q-C has moderate false-positive rates and low PPV, diagnosis of *p*RBD was based on RBD1C questionnaire without PSG confirming, which might result in an overestimation of *p*RBD prevalence in our study. For PD and RBD study, we adopted two-steps of diagnostic procedure and reached the final diagnosis of PD by movement disorders specialists through exclusive investigations. However, it is possible that some mild parkinsonism cases were not detected by local doctors and therefore affected the result of PD-*p*RBD association study.

Overall, we demonstrated that a single question-based questionnaire (RBD1Q-C) was sensitive to screen *p*RBD in large population. Using this tool, we identified the prevalence of *p*RBD was 4.9 % in a rural community and associated with Parkinson’s disease.

## Conclusion

RBD1Q-C was shown to be a useful screening tool with a high sensitivity of 100 % and a moderate specificity of 55.6 %. Based on the RBD1Q-C, we found that the prevalence of *p*RBD was 4.9 % in Chinese rural population aged ≥20 years old and associated with odds of PD, calling for more attention from patients, caregivers and physicians.

## Abbreviations

DLB, lewy body dementia; MSA, multiple system atrophy; MSQ, mayo sleep questionnaire; NPV, negative predictive value; PD, Parkinson’s disease; PPV, positive predictive value; *p*RBD, probable REM sleep behavior disorder; PSG, polysomnographic; RBD, REM sleep behavior disorder; RBD1Q-C, Chinese REM sleep behavior disorder single question; RBD-I, innsbruck RBD inventory; RBDQ-HK, REM sleep behavior disorder screening questionnaire -Hong Kong; RBDSQ, REM sleep behavior disorder screening questionnaire

## Additional file

Additional file 1: RBD single questionnaire. (DOCX 13 kb)

## References

[1] American Academy of Sleep Medicine (2014). International Classification of Sleep Disorders.

[2] Frauscher B, Gschliesser V, Brandauer E (2007). Video analysis of motor events in REM sleep behavior disorder. Mov Disord.

[3] Iranzo A, Molinuevo JL, Santamaria J (2006). Rapid-eye-movement sleep behaviour disorder as an early marker for a neurodegenerative disorder: a descriptive study. Lancet Neurology.

[4] Schenck CH, Bundlie SR, Mahowald MW (1996). Delayed emergence of a parkinsonian disorder in 38 % of 29 older men initially diagnosed with idiopathic rapid eye movement sleep behaviour disorder. Neurology.

[5] Schenck CH, Mahowald MW (2002). REM sleep behavior disorder: clinical, developmental, and neuroscience perspectives 16 years after its formal identification in SLEEP. Sleep.

[6] Chen H, Zhao EJ, Zhang W (2015). Meta-analyses on prevalence of selected Parkinson’s nonmotor symptoms before and after diagnosis. Translational neurodegeneration.

[7] Postuma RB, Arnulf I, Hogl B (2012). A single-question screen for rapid eye movement sleep behavior disorder: a multicenter validation study. Mov Disord.

[8] Frauscher B, Ehrmann L, Zamarian L (2012). Validation of the Innsbruck REM sleep behavior disorder inventory. Mov Disord.

[9] Ohayon MM, Caulet M, Priest RG (1997). Violent behavior during sleep. The Journal of Clinical Psychiatry.

[10] Chiu HF, Wing YK, Lam LC (2000). Sleep-related injury in the elderly--an epidemiological study in Hong Kong. Sleep.

[11] Kang SH, Yoon IY, Lee SD, Han JW, Kim TH, Kim KW (2013). REM sleep behavior disorder in the Korean elderly population: prevalence and clinical characteristics. Sleep.

[12] Stiasny-Kolster K, Mayer G, Schafer S, Moller JC, Heinzel-Gutenbrunner M, Oertel WH (2007). The REM sleep behavior disorder screening questionnaire--a new diagnostic instrument. Mov Disord.

[13] Li SX, Wing YK, Lam SP (2010). Validation of a new REM sleep behavior disorder questionnaire (RBDQ-HK). Sleep medicine.

[14] Boeve BF, Molano JR, Ferman TJ (2011). Validation of the Mayo Sleep Questionnaire to screen for REM sleep behavior disorder in an aging and dementia cohort. Sleep medicine.

[15] Shen SS, Shen Y, Xiong KP (2014). Validation study of REM sleep behavior disorder questionnaire-Hong Kong (RBDQ-HK) in east China. Sleep medicine.

[16] Wang Y, Wang ZW, Yang YC, Wu HJ, Zhao HY, Zhao ZX (2015). Validation of the rapid eye movement sleep behavior disorder screening questionnaire in China. J Clin Neurosci.

[17] Mahlknecht P, Seppi K, Frauscher B (2015). Probable RBD and association with neurodegenerative disease markers: A population-based study. Mov Disord.

[18] Olson EJ, Boeve BF, Silber MH (2000). Rapid eye movement sleep behaviour disorder: demographic, clinical and laboratory findings in 93 cases. Brain.

[19] Dugger BN, Boeve BF, Murray ME (2012). Rapid eye movement sleep behavior disorder and subtypes in autopsy-confirmed dementia with Lewy bodies. Mov Disord.

[20] Bjørnarå KA, Dietrichs E, Toft M (2013). REM sleep behavior disorder in Parkinson’s disease--is there a gender difference. Parkinsonism Relat Disord.

[21] Zhang B, Wing YK (2006). Sex differences in insomnia: a meta-analysis. Sleep.

[22] Postuma RB, Gagnon JF, Vendette M, Fantini ML, Massicotte-Marquez J, Montplaisir J (2009). Quantifying the risk of neurodegenerative disease in idiopathic REM sleep behavior disorder. Neurology.

[23] Iranzo A, Tolosa E, Gelpi E (2013). Neurodegenerative disease status and post-mortem pathology in idiopathic rapid-eye-movement sleep behaviour disorder: an observational cohort study. Lancet Neurology.

[24] Braak H, Del Tredici K, Rub U, de Vos RA, Jansen Steur EN, Braak E (2003). Staging of brain pathology related to sporadic Parkinson’s disease. Neurobiology of aging.

[25] Stiasny-Kolster K, Sixel-Döring F, Trenkwalder C, et al. Diagnostic value of the REM sleep behavior disorder screening questionnaire in Parkinson’s diseas. Sleep Med. 2015;16:186–9. 10.1016/j.sleep.2014.08.014 25534709

