# Supplementary material for: REM sleep behavior disorder was associated with Parkinson’s disease: a community-based study
Source: BMC Neurol. 2016 Aug 2;16:123. doi: 10.1186/s12883-016-0640-1 (PMC4970245; doi:10.1186/s12883-016-0640-1)
Supplement: Additional file 1: — RBD single questionnaire. (DOCX 13 kb) [file 12883_2016_640_MOESM1_ESM.docx]

Could you please answer the following question?

Did you have the following conditions (or have you ever been told by your husband or wife) that you shout, yell, move your arms or legs in response to your dream contents, even fallen off your bed?

1. Yes

0. No

9. Unknown
